# Prevalence and Incidence of Hypoglycaemia in 532,542 People with Type 2 Diabetes on Oral Therapies and Insulin: A Systematic Review and Meta-Analysis of Population Based Studies

**DOI:** 10.1371/journal.pone.0126427

**Published:** 2015-06-10

**Authors:** Chloe L. Edridge, Alison J. Dunkley, Danielle H. Bodicoat, Tanith C. Rose, Laura J. Gray, Melanie J. Davies, Kamlesh Khunti

**Affiliations:** 1 University of Leicester, Department of Health Sciences, Leicester Diabetes Centre, Leicester General Hospital, Leicester, United Kingdom; 2 University of Leicester, Diabetes Research Centre, Leicester Diabetes Centre, Leicester General Hospital, Leicester, United Kingdom; 3 University of Leicester, Diabetes Research Centre, Leicester Diabetes Centre, Leicester General Hospital, Leicester, United Kingdom; 4 University of Leicester, Diabetes Research Centre, Leicester Diabetes Centre, Leicester General Hospital, Leicester, United Kingdom; 5 University of Leicester, Department of Health Sciences, University Road, Leicester, United Kingdom; 6 University of Leicester, Diabetes Research Centre, Leicester Diabetes Centre, Leicester General Hospital, Leicester, United Kingdom; 7 University of Leicester, Diabetes Research Centre, Leicester Diabetes Centre, Leicester General Hospital, Leicester, United Kingdom; University of Tolima, COLOMBIA

## Abstract

**Objective:**

To collate and evaluate the current literature reporting the prevalence and incidence of hypoglycaemia in population based studies of type 2 diabetes.

**Research Design and Methods:**

Medline, Embase and Cochrane were searched up to February 2014 to identify population based studies reporting the proportion of people with type 2 diabetes experiencing hypoglycaemia or rate of events experienced. Two reviewers independently screened studies for eligibility and extracted data for included studies. Random effects meta-analyses were carried out to calculate the prevalence and incidence of hypoglycaemia.

**Results:**

46 studies (n = 532,542) met the inclusion criteria. Prevalence of hypoglycaemia was 45% (95%CI 0.34,0.57) for mild/moderate and 6% (95%CI, 0.05,0.07) for severe. Incidence of hypoglycaemic episodes per person-year for mild/moderate and for severe was 19 (95%CI 0.00, 51.08) and 0.80 (95%CI 0.00,2.15), respectively. Hypoglycaemia was prevalent amongst those on insulin; for mild/moderate episodes the prevalence was 50% and incidence 23 events per person-year, and for severe episodes the prevalence was 21% and incidence 1 event per person-year. For treatment regimes that included a sulphonylurea, mild/moderate prevalence was 30% and incidence 2 events per person-year, and severe prevalence was 5% and incidence 0.01 events per person-year. A similar prevalence of 5% was found for treatment regimes that did not include sulphonylureas.

**Conclusions:**

Current evidence shows hypoglycaemia is considerably prevalent amongst people with type 2 diabetes, particularly for those on insulin, yet still fairly common for other treatment regimens. This highlights the subsequent need for educational interventions and individualisation of therapies to reduce the risk of hypoglycaemia.

## Introduction

Hypoglycaemia in type 2 diabetes is associated with a considerable cost and burden to the health service, with an estimated annual cost to the NHS of £39 million[1]. There can also be substantial consequences for the individual, with an increased risk of mortality and morbidity from severe episodes [2–4]. Hypoglycaemia significantly impacts on an individual’s quality of life, their employment, social interactions, and driving [5–7]. In addition to the direct effects of hypoglycaemia, there may be a substantial indirect impact on serious long-term health consequences from medication non-adherence and purposeful hyperglycaemia, due to fear and avoidance of hypoglycaemia [8].

A common cause of hypoglycaemia is iatrogenic [9]. In order to avoid long-term complications of type 2 diabetes, emphasis is placed on improving blood glucose control [9–11]. A recent meta-analysis revealed that intensive glycaemic control in people with type 2 diabetes can result in a 17% reduction in non-fatal myocardial infarction and a 15% reduction in coronary heart disease events [12]. To help achieve tight glycaemic control, people with type 2 diabetes are frequently placed on intensive treatment regimens, including earlier initiation of insulin. Intensive regimens and tighter glycaemic control have been shown to increase the risk of hypoglycaemia[13–15]. However, the topic of glycaemic management and pharmacological treatments is becoming more complex. Newer therapies and more treatment combinations, are increasingly becoming available, with the aim of maximising glucose control without the increased risk of hypoglycaemia[9, 16].

In addition to treatment regimes, other currently identified potential risk factors for hypoglycaemia in type 2 diabetes include exercise [17], increased age [18], presence of co–morbidities [18], hypoglycaemia unawareness [18], dietary mistakes [19], excessive dieting [20] or weight loss, alcohol [21], number of years since diabetes diagnosis [22], and time since insulin initiated [23],

Hypoglycaemia prevalence in real world type 2 diabetes settings has been considered [3, 24], however, there has not been a systematic review and meta-analyses of the literature. Previously published systematic reviews that have considered hypoglycaemic episodes in type 2 diabetes have tended to focus on clinical trials of the safety and efficacy of a particular drug [15, 25–30]. Clinical trials usually exclude participants at higher risk of hypoglycaemia, attract more motivated and selective participants, have a treat to target design and place participants on treatment regimens specifically for the study. Consequently, generalisability of findings to real world settings may be limited and hypoglycaemia prevalence and incidence in clinical trials may be lower than in clinical practice. Knowing the incidence of hypoglycaemia is important to provide insight into its impact both clinically and from a patient level. It enables the planning of resources, exploration of risk factors and design of interventions for prevention of hypoglycaemia. Additionally, the frequency and severity of hypoglycaemia is often used as a rationale for the use of newer treatments and as a clinical indicator for the choice of treatment patients are placed on.

To our knowledge, there have been no systematic reviews of the prevalence or incidence rates of hypoglycaemic events in type 2 diabetes in population based studies. This systematic review aimed to collate and evaluate the current literature reporting the prevalence (proportion of people) and the incidence (rate of episodes) of hypoglycaemia in a real world type 2 diabetes population.

## Methods

### Search Strategy and Study Selection

We searched electronic bibliographic databases Ovid Medline (including in-process and other non-indexed citations) and Embase from 1998 to February 2014 and Cochrane (issue 2, 2014), using a combination of keywords and MeSH terms with English and American spellings. The search terms used covered type 2 diabetes, hypoglycaemia prevalence and hypoglycaemia incidence. An example search strategy tailored for Ovid medline can be found in S1 Fig.

The primary aim of this systematic review was to explore the incidence and prevalence of hypoglycaemia in people with type 2 diabetes within a general population-based setting. We included observational studies where: 1) the study population were a defined general population sampled from either a defined geographical location, attendees at a primary, secondary or other healthcare centre, or people registered on a health service or health insurance database; 2) the study population (or sub-population) all had type 2 diabetes and were aged ≥ 18 years old; 3) they were published in English language; and 4) they were published as full papers. We additionally required studies to report the number of type 2 diabetes participants who had experienced ≥ 1 hypoglycaemic episode, the incidence of hypoglycaemic episodes experienced, or data to allow the calculation of one of these measures. We did not apply any restrictions relating to the classification, definition or measurement of how hypoglycaemia was utilised by studies. We excluded studies if: 1) they were pharmacological trials or the study methods involved any alteration to a participant’s treatment or care, either pharmacological or behavioural; 2) the majority of participants were pregnant, fasting, on a restrictive diet, or were selected on the basis of having a specific acute or chronic illness; 3) participants were selected on the basis of their hypoglycaemia history or recent initiation of treatment regime; 4) participants were sampled via an established survey/consumer panel or a consumer database; or 5) hypoglycaemia rates were solely reported over less than one week.

Following removal of duplicate publications, titles and abstracts were reviewed independently by two reviewers (CE, TR) in order to identify studies that met the inclusion criteria. Where it was unclear from the abstract whether the inclusion criteria were met, the full article was retrieved and reviewed. In instances where there were disagreements between reviewers, a third reviewer (AJD or KK) was consulted.

### Data extraction

A data extraction form was developed and pilot tested, with adaptations made accordingly. Two reviewers (CE, TR) extracted independently from included studies.

Where data were available, we extracted the following for each study: 1) study details (including study design, year published and country); 2) population details (including sample source, mean age, ethnicity, mean HbA1c, sex,treatment regimens and cardiovascular desease or events); 3) methods used to measure hypoglycaemia (self-report questionnaires, prospective diaries, emergency department admission records, claims databases); 4) the time period hypoglycaemia was measured over; and 5) severity measured (definition given by the study). We extracted outcome data for the proportion of T2DM participants only who had experienced at least one hypoglycaemic episode (prevalence) and the incidence rate of episodes experienced (or data to calculate).

### Quality assessment

We created a quality assessment tool using elements from the Effective Public Health Practice Project (EPHPP) Quality Assessment Tool for Quantitative Studies [31], the Cochrane Collaboration Risk of Bias Tool [32] and the Quality assessment tool for systematic reviews of Observational Studies (QATSO) [33], see S1 Table. Two reviewers (CE, TR) independently assessed all studies for methodological quality. Each criterion was given a score of ++, + or-, and an overall quality grade was assigned for each of the following; sample bias (sample source representative and described well, sampling method, eligibility criteria applied and described, sufficient sample response), data collection bias (data collection tool well described, measurement reliability) and confounding and explanatory factors considered.

### Data synthesis

For analytic and descriptive purposes, we categorised and defined hypoglycaemia by severity of the episode measured within the study. Where possible, based on the description given in the paper, studies were categorised as either mild/moderate or severe. Mild/moderate hypoglycaemia was referred to by studies when no third party assistance was needed during a hypoglycaemic episode. Severe hypoglycaemia was used by studies when third party assistance was required. However, some studies did not specify a severity when gathering some or all of the hypoglycaemia data; these data were classified as “unspecified” hypoglycaemia. It was assumed that the “unspecified” data covered both severe and mild/moderate hypoglycaemia. Where a study reported data separately for more than one of the classifications (severe, mild/moderate and unclassified), we extracted and analysed the data separately. If a study reported zero hypoglycaemic events, we entered 0.001 into the meta-analysis to avoid the study being excluded [32]. Where two prevalence or incidence means were reported in a paper for different populations, the pooled mean was calculated [32]. Population treatment categories relating to hypoglycaemia prevalence and incidence, were based on the available data. Where possible, data were grouped into: insulin, with or without oral-glucose lowering therapies; sulphonylureas, non-insulin but with or without other oral-glucose lowering therapies; non-sulphonylureas, non-insulin and non sulphonylurea oral-glucose lowering therapies; or mixed oral-glucose lowering therapies, non-insulin but no further description given relating to type of oral-glucose lowering therapies. For studies where treatment categories were not mutually exclusive, if the whole population were pharmacologically controlled, data for insulin were subtracted from the overall data, where possible, and the remaining data were grouped in the mixed oral glucose lowering category.

For the primary outcome of interest (the proportion of people who had experienced hypoglycaemia), we conducted meta-analyses for mild/moderate episodes, severe episodes and unspecified episodes. We carried out sub-analyses within these categories by treatment regime of the population. We also carried out a sub-analysis for severe hypoglycaemia which specifically required emergency or medical assistance. We used meta-regression techniques to assess the following potential explanatory variables at the study-level: mean age, percentage of male participants, mean HbA1c level, and the time over which hypoglycaemia was measured.

We carried out further meta-analyses for the rate of hypoglycaemic episodes per person year, for the three categories of, mild/moderate, severe and unspecified. Again, analyses were stratified by therapy option.

When calculating confidence intervals for meta-analyses, negative values can be found. We decided to cap all confidence intervals at 0.00, due to it not being plausible to have a negative confidence interval for prevalence or incidence.

Heterogeneity was assessed using the I^2^ statistic. Due to high levels of heterogeneity, we used random-effects models throughout to calculate effect sizes. Publication bias was assessed with a funnel plot and the Egger test. This was carried out separately for studies reporting, mild/moderate, severe and unspecified events and separately for prevalence and incidence. Significance was set at P<0.05, all p-values are two-sided and 95% confidence intervals are quoted throughout. We performed all analyses in Stata version 13 (StataCorp, College Station, TX).

## Results

### Identification of studies

**Fig 1:**
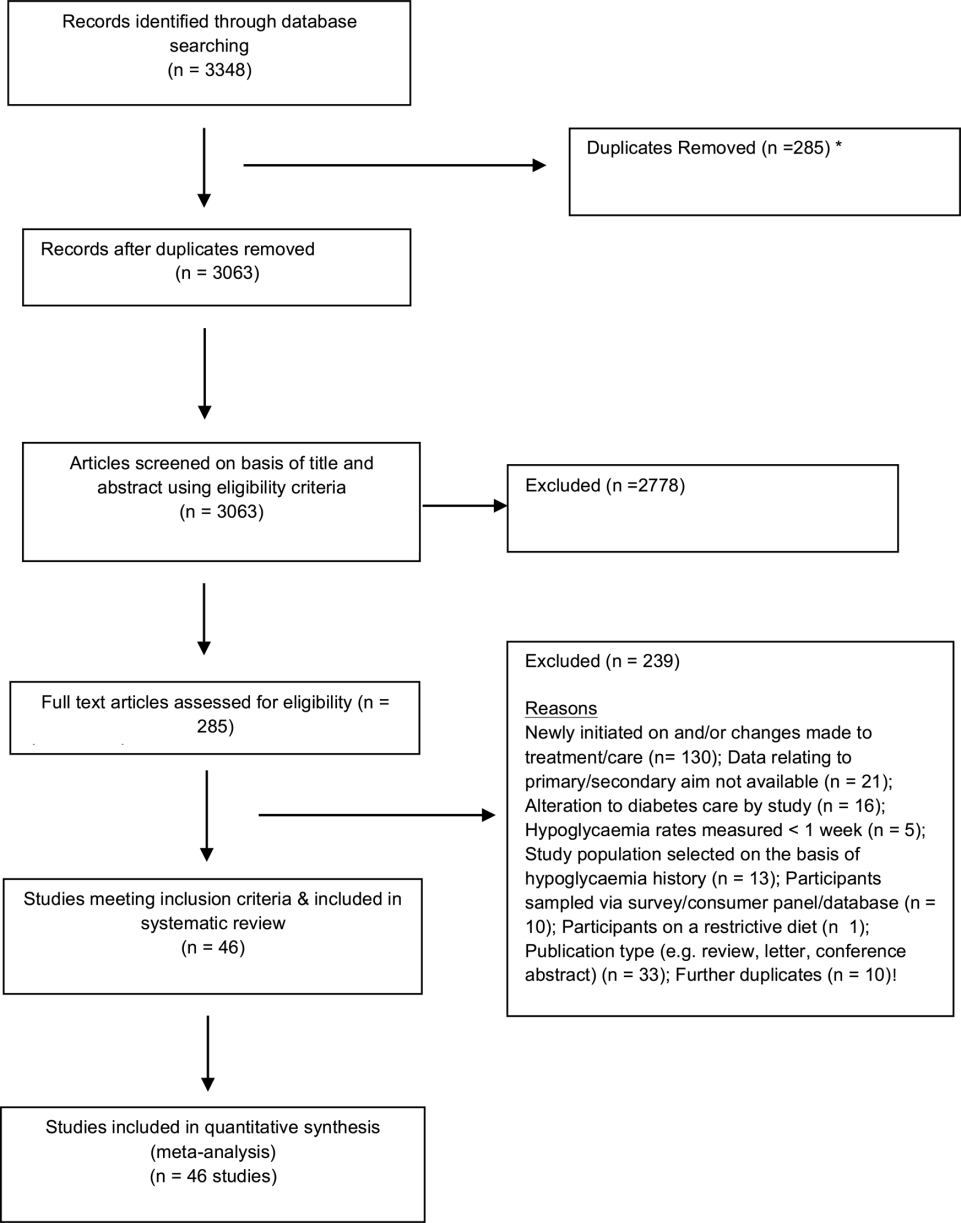
Flowchart of selection of studies from search to final inclusion.

Results relating to the identification process for eligible studies are summarised in Fig 1. Searches yielded 3348 citations, and 3063 unique titles and abstracts were screened for eligibility. Following full text retrieval of 285 potentially relevant papers, 239 were subsequently excluded, leaving 46 papers eligible for inclusion in the analyses.

### Summary of included studies

Descriptive characteristics including the definitions and descriptions of hypoglycaemia used bythe 46 studies included in the systematic review are summarised in Table 1. All studies were observational, with 27 being cross-sectional, 11 prospective, 6 retrospective and 2 used a mixed methods design of cross-sectional/prospective (n = 1) or cross-sectional/retrospective (n = 1). Papers included were published from 1998 to 2013 inclusive. The number of participants within each study ranged from 41 to 361,210. Studies were conducted in Europe (n = 25), North America (n = 14), Australia (n = 2), and Eastern/South East Asia (n = 5). Ethnicity was poorly reported; of the 9 (19.6%) studies that reported ethnicity, the proportion of non-white participants ranged from 17% to 100%. Population samples were obtained from either health clinic attendees (n = 21), diabetes registries/databases (n = 8), health insurance databases (n = 7), emergency department records (n = 4), community populations (n = 3) or pharmacy records (n = 2).

**Table 1:** Characteristics of studies included in the systematic review.

**First author (year)**	Data Source (Hypo info)	Country	Sample size	Mal %	Mean Hba1c% mmol	CVD events Hypo: No hypo	Non-white %	Mean Age (years)	Sampling method	Sample source	Design	Length of therapy (years)	Time hypo measured over	Hypo definition	Analysis^d^
Akram 2006	Questionnaire/ Recall	Denmark	401	58	8.3 (67.2) (median)		NR	66 (median)	Consecutive	Health clinic attendees (outpatients diabetes clinic, single centre)	Cross-sectional	7 (median) (insulin)	1 Year	Need for assistance	Severe^b^
Allen 2004	Prospective Diary	UK	41	63.4	NR		NR	NR	Consecutive	Health clinic/centre (outpatients, single centre)	Prospective	1.4 (mean) (insulin)	3 Months	Required glucose/glucogen. If tested BG <3.5mmol/L	Mild^a^, Severe^b^
Andel 2008	Questionnaire/ Recall/Diary	Central/ Eastern europe	8,231	47.3	7.7 (60.7)		NR	62.2	Consecutive	Health clinic attendees (secondary care outpatient diabetes centres, multicentre)	Cross-sectional	NR	1 Year	Local clinical standards + guidelines	Severe^b^
Aung 2011	Questionnaire/ Recall	UK	10,66	51	7.3 (56.3)	Myocardial infaraction 24%:13% Angina 44%:26% Stroke 10%:5% transient ischaemic attack 5%:24%	NR	67.9	Random	Diabetes registry/database (1 geographical location)	Cross-sectional	NR	1 Year	Need for assistance	Severe^b^
Bourdelmarc-hasson 2007	Questionnaire/ Recall	France	2,832	60.6	7.1 (54.1)		NR	63.8	Random	Health insurance database (national)	Cross-sectional	NR	1 Year	Need for assistance	Severe^b^
Chan 2010	Questionnaire/ Recall	Asia	2,257	49.4	7.5 (58.5)		NR	NR	Consecutive	Health clinic attendees (outpatients, multicentre)	Cross-sectional	NR	6 Months	Level of assistance/interruption to activities	Unspecified^c^, Mild^a^, Severe^b^
Davis 2005	Questionnaire/ Recall	UK	590	58	NR		NR	NR	Stratified	Health clinic records or from previous study	Cross-sectional	Not reported for the study cohort included for hypo analysis	3 Months	Level of Symptoms, behvaiour, assistance	Unspecified^c^, Mild^a^, Severe^b^
Davis 2010	Questionnaire/ Recall	Australia	616	52.3	NR		NR	67	Non-random	Community population (particular geographical location)	Prospective	NR	6.4 months (Mean)	Diagnosed by health service	Severe^b^
Donnelly 2004	Prospective Diary	UK	173	53	8.9 (73.8)		NR	65	Random	Diabetes registry/database (1 geographical location)	Prospective	NR	1 Month	Need for assistance	Unspecified^c^, Mild^a^, Severe^b^
Green 2012	Questionnaire/ Recall	USA	3,000	40.4	NR	Heart Disease 31%:22%	NR	NR	Stratified random	Community population (postcode defined)	Cross-sectional	NR	1 Year	Low BG	Unspecified^c^
Gurlek 1999	Questionnaire/ Recall	Turkey	114	44.7	NR		NR	58.9	Unclear	Health clinic attendees (outpatients, single centre)	Retrospective	NR	3.33 years (mean)	Help needed due to neuroglycopenia/required parenteral glucose	Severe^b^
Henderson 2003	Questionnaire/ Recall	UK	215	NR	8.6 (70.5)		NR	NR	Random	Health clinic attendees (outpatients, diabetes clinic, single centre)	Cross-sectional	NR	1 Year	Required glucose/glucagon, level of assitance	Unspecified^c^, Mild^a^, Severe^b^
Holstein 2003	Emergency records	Germany	9,000	NR	NR		NR	NR	All patients included that met inclusion criteria	Emergency records from one geographical location	Prospective	NR	4 Years	Required intravenous glucose/glucagon. Confirmed BG.	Severe^b^
Honkasalo 2011	Questionnaire/ Recall/ Emergency dep records	Finland	1,065	NR	NR		NR	NR	All participants eligible invited	Health insurance database (2 geographical locations)	Cross-sectional/ Retrospective	NR	1 Year	Need for assistance	Severe^b^
Jaap 1998	Questionnaire/ Recall	UK	132	NR	NR		NR	NR	Consecutive	Health clinic attendees (outpatients, diabetes clinic, multicentre)	Cross-sectional	NR	2 Months	Symptoms resolved with glucose/glucagon	Unspecified^c^
Johnston 2012	Claims database	USA	361,210	51.6	NR	Iscaemic heart disease 26%:19% Congestive heart failure 12%:6% Myocardial infaraction 2%:1% Angina 2%:1% Transient ischaemic attack 7%:5%	NR	NR	All patients included that met inclusion criteria	Health insurance database	Retrospective	NR	1 Year	Need for assistance	Severe^b^
Katon 2013	Emeregncy dep records/Health insurer	USA	4,117	51.9	NR		19.9	63.4	All participants eligible invited	Diabetes registry/database (primary care centres)	Prospective	NR	5 years	Emergency visit or hospitalisation	Severe^b^
Krnacova 2012	Emergency records	Czeh Republic	37,459	NR	NR		NR	NR	All patients included that met inclusion criteria	Emergency records from one geographical location	Prospective	NR	1 Year	Assistance of emergency services	Severe^b^
Lecomte 2008	Questionnaire/ Recall	France	3,324	53.8	NR		NR	NR	Random	Health insurance database (national)	Cross-sectional	NR	2 Year	Need for assistance	Severe^b^
Leese 2003	Emergency records	UK	7,678	52	NR		NR	65.8	All patients included that met inclusion criteria	Diabetes registry/database (1 geographical location)	Prospective	NR	3 Year	BG <3.5 mmol/L-glucose/glucagon needed	Severe^b^
leiter 2005	Questionnaire/ Recall	Canada	133	NR	7.52 (58.5)		NR	59.9	Convenience	Health clinic attendees (multicentre)	Cross-sectional	10.2 (mean) (insulin)	1 month/1 year	BG <4.0mmol/L or <2.8 with assistance	Mild^a^, Severe^b^
Lin 2012	Claims database/ Health insurer	Taiwan	15,404	45.1	NR	Iscaemic heart disease 27%:15% Cardiovascular disease 83%:63%	100	64.2	Random	Health insurance database (national)	Retrospective	NR	2 Years	Medical assistance required	Severe^b^
Lipska 2013	Questionnaire/ Recall	USA	9,094	50	7.5 (58.5)	Congestive heart failure 14%:6% Cerebrovascular disease 9%:4% Myocardial infaraction 9%:6%	74.3	59.5	Stratified random	Diabetes registry/database (1 geographical location)	Cross-sectional	NR	1 Year	Need for assistance	Severe^b^
Lundkvist 2005	Questionnaire/ Recall	Sweden	309	60	NR	Macro (mean complications) 0.24:0.25	NR	65	Convenience	Health clinic attendees (7 centres)	Cross-sectional	NR	2 Year	Level of assistance or BG <3.3 mmol/L	Unspecified^c^, Severe^b^
Maggi 2013	Questionnaire/ Recall	Italy	1,342	52.5	7.2 (55.2)		NR	73.3	Consecutive	Health clinic attendees (57 diabetes centres)	Cross-sectional	NR	3 Months	Requiring hospitalisation	Unspecified^c^, Severe^b^
McCoy 2012	Questionnaire/ Recall	USA	797	NR	NR		NR	NR	Convenience	Health clinic attendees (diabetes clinic, single centre)	Cross-sectional	NR	6 Months	Level of symptoms, assistance	Severe^b^
McCoy 2013	Questionnaire/ Recall	USA	326	53.1	7.6 (59.6)		NR	67.1	All patients included that met inclusion criteria	Diabetes registry/database (1 geographical location)	Cross-sectional		6 Months	Level of symptoms, assistance	Severe^b^
Miller 2001	Questionnaire/ Recall	USA	1,055	28.2	7.6 (59.6)		93.8	60.9	All patients who met inclusion criteria and had sufficient data included	Health clinic attendees (diabetes clinic, single centre)	Cross-sectional	NR	3.17 Months (mean)	Symptoms, behaviour, need for assistance or glucose <3.3mmol/L	Unspecified^c^, Severe^b^
Murata 2004	Prospective Diary	USA	344	NR	NR		NR	NR	Random	Pharmacy records (3 centres)	Prospective	8.1 (insulin) (mean)	11 Months (mean)	BG<3.3mmol/L	Unspecified^c^
Neil 2007	Questionnaire/ Recall	USA	5,965	NR	NR		NR	NR	All participants eligible invited	Pharmacy records (5 centres)	Cross-sectional	NR	6 Months	Need for assistance	Unspecified^c^, Severe^b^
Ooi 2011	Questionnaire/ Recall	Malaysia	170	41.2	8.02 (64.2)		100	NR	Consecutive	Health clinic attendees (primary care, 7 centres)	Cross-sectional	NR	1 Year	Symptoms	Unspecified^c^
Parsaik 2013	Emergency records	USA	5,534	NR	NR		NR	NR	All patients included that met inclusion criteria	Emergency records from one geographical location	Retrospective	NR	6 years	Assistance of emergency services	Severe^b^
Pettersson 2010	Questionnaire/ Recall	Sweden	430	61	6.3 (45.4)	Macrovascular events 33%:32%	NR	69	Consecutive	Health clinic attendees (primary care, multicentre)	Cross-sectional	NR	6 Months	Level of assistance/interruption to activities	Unspecified^c^, Severe^b^
Rombopoulos 2013	Questionnaire/ Recall	Greece	6,631	55	NR		NR	NR	Random	Community population (defined geographical locations distributed)	Cross-sectional	NR	3 Months	Laboratory-confirmed clinical symptomatic	Unspecified^c^, Severe^b^
Samann 2012	Questionnaire/ Recall/ Emergency dep records	Germany	4,481	56	NR		NR	66	All participants eligible invited	Health insurance database	Retrospective	NR	1 Year	Coma or glucose/glucagon required	Severe^b^
Sarkar 2010	Questionnaire/ Recall	USA	14,357	51	7.6 (59.6)		78	58	Stratified random	Health insurance database	Cross-sectional	NR	1Year	Passing out or need for assistance	Severe^b^
Schopman 2009	Questionnaire/ Recall/ Prospective diary	UK	110	51.6	NR		NR	NR	Random	Health clinic attendees (secondary care, diabetes clinic, single centre)	Cross-sectional/ Prospective	6.5 (median) (insulin)	1 month/1 year	Need for assistance or BG <3.3 mmol/L	Severe^b^
Skinner 2013	Questionnaire/ Recall	Australia	1,926	NR	NR		NR	NR	Random	Diabetes registry/database (national)	Cross-sectional	NR	1 week	Low BG	Unspecified^c^
Stahl 1999	Emergency records	Switzerland	2,529	NR	NR		NR	NR	All patients included that met inclusion criteria	Emergency records from one geographical location	Retrospective	NR	1 Year	Hospital Admission	Severe^b^
Stargardt 2009	Questionnaire/ Recall	Germany	392	57.4	7.2 (55.2)		NR	62.7	Convenience	Health clinic attendees (92 centres)	Cross-sectional	NR	6 Months	According to level of assistance/interruption to activities	Unspecified^c^, Severe^b^
Vexiau 2008	Questionnaire/ Recall	France	400	53.6	7.2 (55.2)	Macrovascular events 26%:17%	NR	62.1	Non-random	Health clinic attendees (primary care, 98 centres)	Cross-sectional	NR	7 Months	According to level of assistance/interruption to activities	Unspecified^c^, Mild^a^, Severe^b^
Whitmer 2009	Emergency records	USA	16,667	54.6	NR	Heart disease 84%:62% Stroke 44%:29%	37.2	64.9	All participants eligible invited	Diabetes registry/database	Prospective	6.6 (mean) (insulin	22 years	Emergency department diagnosis	Severe^b^
Williams 2012	Questionnaire/ Claims database	USA	813	58	NR		17	57	Random	Health insurance database	Cross-sectional	NR	1 Year	Symptoms or BG <70mg/dL	Unspecified^c^, Severe^b^
Yun 2013	Questionnaire/ Recall/ Emergency dep records	Korea	878	38	8.8 (72.7)		NR	55.3	Consecutive	Health clinic attendees (outpatients, single centre)	Prospective	NR	10.42 years (median)	Assistance needed to actively administer carbohydrate	Severe^b^
The UK Hypoglycaemia study group 2007	Prospective diary	UK	274	70.1	7.5 (58.5)		NR	61	Stratified	Health clinic attendees (secondary diabetes, 6 centres)	Prospective	not reported for the study cohort included for hypo analysis	10.50 months (median	Symptoms or glucose BG <3.0 mmol/l + according to level of assistance	Mild^a^, Severe^b^
Zhang 2013	Questionnaire/ Recall	China	586	59.2	7.5(58.5)		100	55.1	Consecutive	Health clinic attendees (secondary care, outpatients, single centre)	Cross-sectional	NR	3 Months	NR	Unspecified^c^

Abbreviations; HbA1c - glycated haemoglobin, NR—not reported, BG—blood sugar, Hypo–hypoglycaemia.

^a^ Mild/moderate hypoglycaemia—no third party assistance was needed

^b^ Severe hypoglycaemia—third party assistance was required.

^c^ Unspecified hypoglycaemia- severity not specified when gathering some or all of the hypoglycaemia data

^d^ Category for meta-analysis

Over half of the studies reported only severe hypoglycaemia (n = 24), while 3 studies reported severe and mild/moderate. There were 6 studies which reported data on unspecified hypoglycaemia, 8 which reported data on unspecified hypoglycaemia along with severe, and 5 which reported data on unspecified, mild/moderate and severe. For studies reporting the prevalence of hypoglycaemia, the time period used for recall of previous hypoglycaemic episodes ranged from 1 month to 22 years. The majority of studies (n = 17) measured episodes over a period of 12 months. The measurement period for studies reporting the incidence of hypoglycaemia, ranged from 1 week to 22 years. The most frequently used was 12 months (9/21). A variety of methods/sources, either alone or in combination, were utilised to obtain relevant data. These included questionnaires (n = 25), emergency department records (n = 7), prospective diaries (n = 4), and claims databases (n = 3).

### Study quality

A breakdown of study quality is presented in S1 Table. Most studies received a high quality grading for the consideration of data collection bias (42/46, 91.3%) and confounding and explanatory factors (43/46, 93.5%). However, under half of studies scored well for sample bias (15/46, 32.6%).

### Prevalence of people who have experienced hypoglycaemia

**Fig 2:**
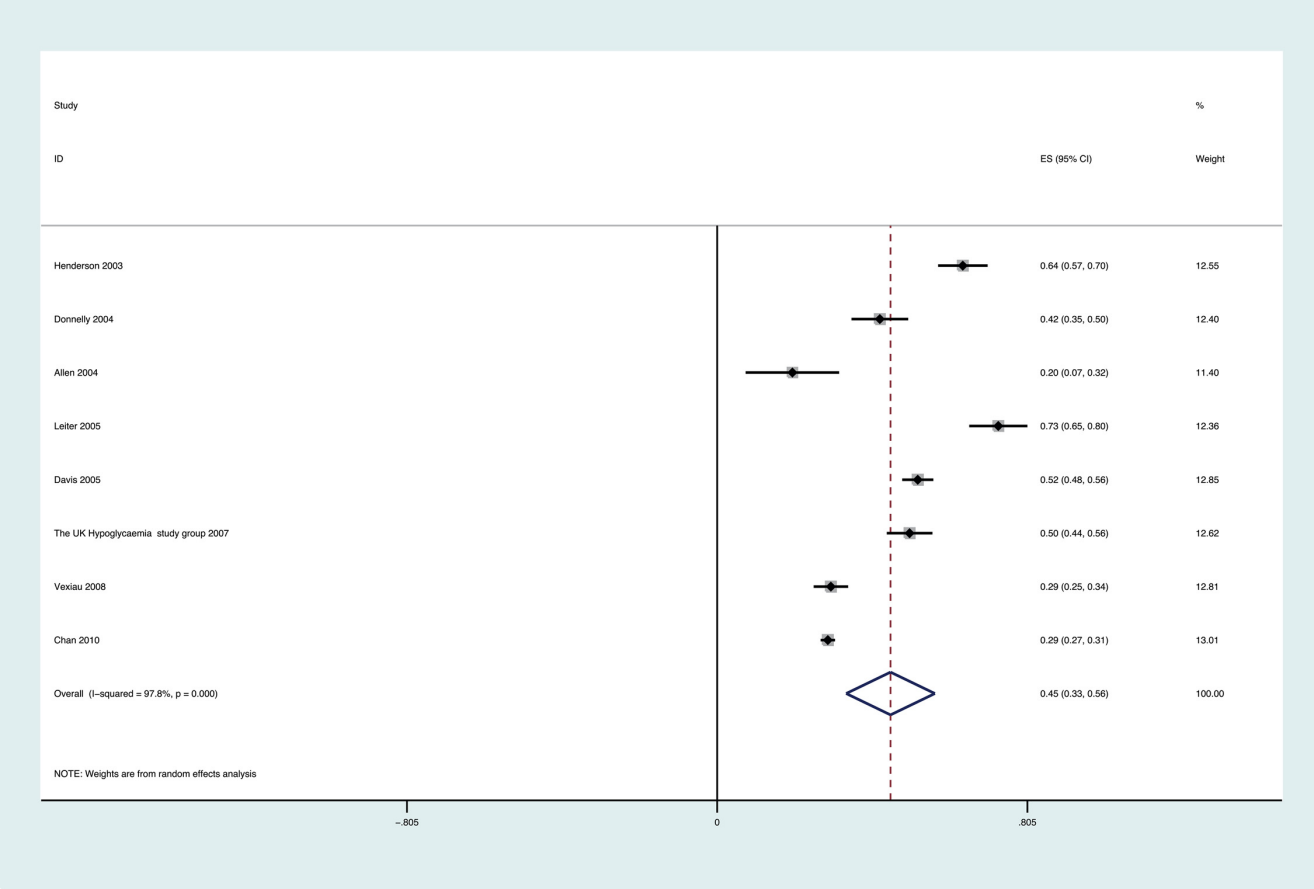
Forest plot showing the proportion of people experiencing mild/moderate hypoglycaemia in each study and the overall pooled estimate. Boxes and horizontal lines represent proportion of people experiencing unspecified hypoglycaemia and the corresponding 95% CI, respectively, for each study. Size of box is proportional to weight of that study result. Diamonds represent the 95% CI for pooled estimates of effect and are centred on pooled hypoglycaemia incidence.

Overall 46 studies involving 532,542 participants were included for the meta-analyses examining the prevalence (proportion of people who had experienced hypoglycaemia). Eight papers involving 4,083 participants, measuring over a period of 1 month to 10.5 months, reported mild/moderate hypoglycaemia. The pooled prevalence of people who had experienced hypoglycaemia was 0.45 (95% CI 0.34 to 0.57; Fig 2). In relation to those on insulin as a diabetes treatment regime, the prevalence was 0.52 (95% CI 0.37 to 0.67) compared with 0.33 (95% CI 0.24 to 0.42) for sulphonylureas. Data were not available to calculate any further treatment categories.

**Fig 3:**
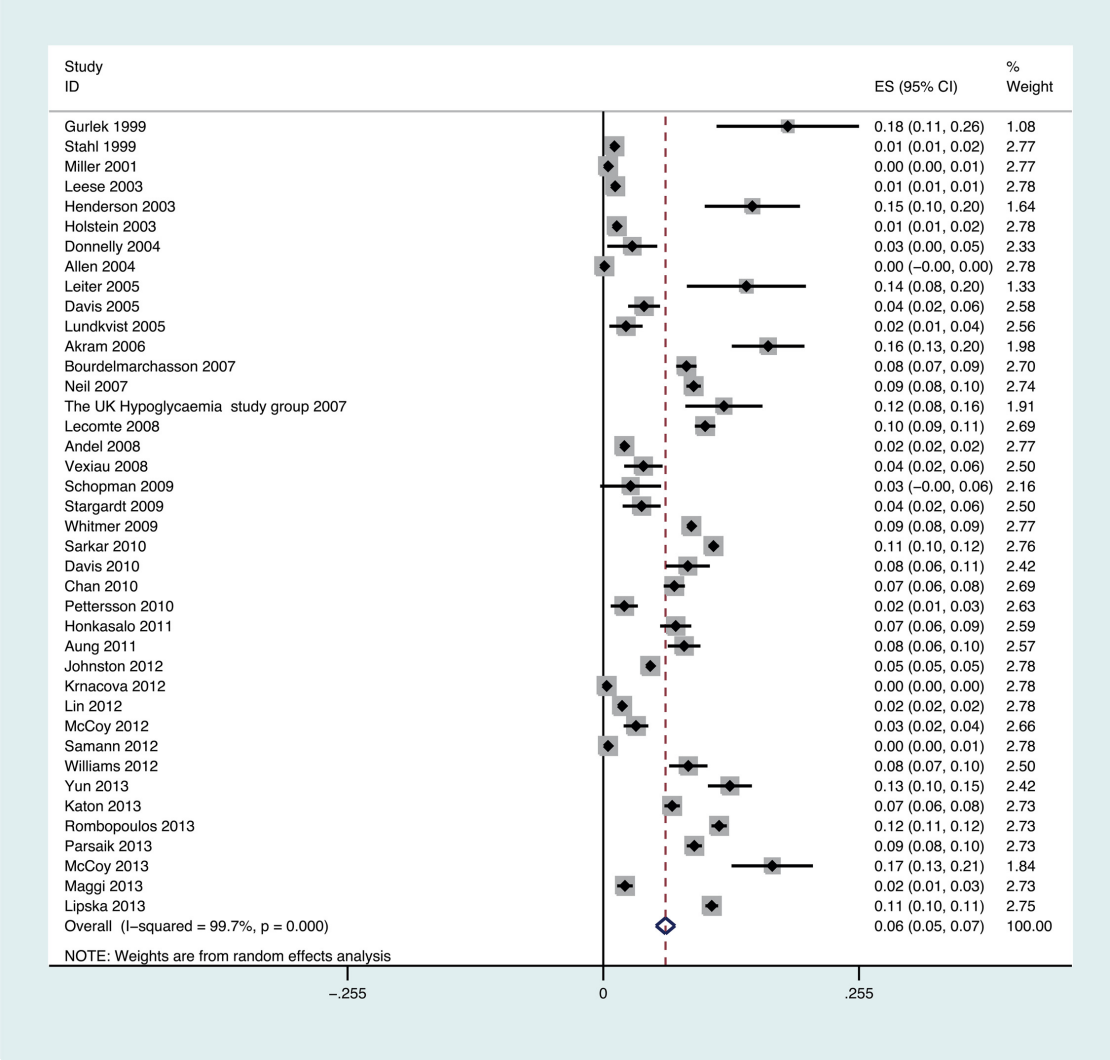
Forest plot showing the proportion of people experiencing severe hypoglycaemia in each study and the overall pooled estimate. Boxes and horizontal lines represent proportion if people experiencing severe hypoglycaemia and 95% CI for each study. Size of box is proportional to weight of that study result. Diamonds represent the 95% CI for pooled estimates of effect and are centred on pooled hypoglycaemia incidence.

The meta-analysis for the prevalence of severe hypoglycaemia included 40 papers involving 528,310 participants, measuring over a period of 1 month to 22 years, with a pooled prevalence of 0.06 (95% CI 0.05 to 0.07; Fig 3). For people on insulin as a treatment regime, the prevalence was relatively higher at 0.21 (95% CI 0.16 to 0.25) when compared with regimens involving sulphonylurea (0.05 [95% CI 0.02 to 0.07]), non sulphonylurea therapies (0.05 [95% CI 0.03 to 0.07] and mixed oral glucose lowering therapies (0.05 [95% CI 0.02 to 0.07]); Table 2). In 14 studies involving 473,481 participants, severe hypoglycaemic episodes were restricted to only those requiring medical assistance (not any third party assistance), the overall pooled prevalence was 0.05 (95% CI 0.03 to 0.06). When these studies were excluded the overall pooled prevalence for severe hypoglycaemia was 0.08 (95% CI 0.06 to 0.10), see Table 2.

**Table 2:** Proportion of people experiencing hypoglycaemia and the number of episodes per patient-year by severity and treatment regime.

	Unspecified		Mild/Moderate		Severe	
	Proportion	Rate	Proportion	Rate	Proportion	Rate
**Insulin** ^**a**^						
N studies (subjects)	8 (2539)	3 (649)	5 (728)	3 (513)	20 (17881)	11 (6851)
Pooled estimate (95% CI)	0.57 (0.42 to 0.71)	9.25 (1.38 to 17.13)	0.52 (0.37 to 0.67)	23.31 (0.00 to 58.98)	0.21 (0.16 to 0.25)	1.05 (0.00 to 3.69)
I^2^	98.4%	99.7%	94.5%	100.0%	99.7%	92.8%
**Sulphonylureas** ^**b**^						
N studies (subjects)	6 (8390)	0 (0)	2 (508)	1 (108)	8 (12872)	3 (3538)
Pooled estimate (95% CI)	0.26 (0.02 to 0.50)	Insufficient data	0.33 (0.24 to 0.42)	1.92 (0.51 to 3.33)	0.05 (0.02 to 0.07)	0.01 (0.00 to 0.55)
I^2^	98.4%		69.1%	N/A	98.5%	0.0%
**Non-sulphonylureas** ^c^						
N studies (subjects)	4 (2135)	0 (0)	0 (0)	0 (0)	2 (646)	0 (0)
Pooled estimate (95% CI)	0.26 (0.02, to 0.50)	Insufficient data	Insufficient data	Insufficient data	0.05 (0.03 to 0.07)	Insufficient data
I^2^	99.6%				0.0%	
**Mixed** oral-glucose lowering therapies ^d^						
N studies (subjects)	2 (568)	0 (0)	0 (0)	0 (0)	7 (35041)	2 (1861)
Pooled estimate (95% CI)	0.40 (0.30 to 0.51)	Insufficient data	Insufficient data	Insufficient data	0.05 (0.02 to 0.07)	0.01 (0.00 to 5.16)
I^2^	0.0%				99.5%	0.0%
**Proportion overall** ^e^						
N studies (subjects)	18 (24804)	5 (5575)	8 (4083)	4 (621)	40 (528310)	19 (76254)
Pooled estimate (95% CI)	0.43 (0.36 to 0.50)	27.78 (0.00 to 58.20)	0.45 (0.34 to 0.57)	19.03 (0.00 to 51.08)	0.06 (0.05 to 0.07)	0.80 (0.00 to 2.15)
I^2^	99.4%	100.0%	97.8%	100.0%	99.7%	87.0%

Abbreviations: CI, Confidence Interval.

^a^ Insulin (with or without oral-glucose lowering therapies)

^b^ Sulphonylureas (non-insulin but with or without other oral-glucose lowering therapies)

^c^ Non-sulphonylureas (non-insulin and non-sulphonylurea oral-glucose lowering therapies)

^d^ Mixed oral-glucose lowering therapies (non-insulin but no further description given relating to type of oral-glucose lowering therapies).

^e^ All studies combined regardless of treatment

Eighteen papers involving 24,804 participants, measuring over 1 month to 1 year, reported data for the prevalence of unspecified type of hypoglycaemia (severity of hypoglycaemia experienced was not specified when data collecting, assumed to cover both mild/moderate and severe episodes), with a pooled prevalence of 0.43 (95% CI 0.36 to 0.50).

There was high heterogeneity shown between studies reporting mild/moderate (I^2^ = 98.0%), severe (I^2^ = 99.7%) unspecified (I^2^ = 99.4%) hypoglycaemia prevalence. A possible explanatory variable for this was the different time periods hypoglycaemia was measured over between studies. This along with other explanatory variables: year of publication, mean HbA1c, mean age, and percentage of male participants were carried out in a meta-regression on the prevalence of, mild/moderate, severe and unspecified hypoglycaemia. No variables were shown to be statistically significant, see Table 3.

**Table 3:** Meta-regression results showing the effect of study-level variables on the proportion of people experiencing each severity of hypoglycaemia.

Explanatory variable	N studies	Effect (95% CI)	P-value
**Unspecified Hypoglycaemia**			
Mean age, years	14	0.00 (0.00, 0.00)	0.52
% Male participants	9	0.00 (0.00, 0.00)	0.31
Mean HbA1c, %	10	0.15 (0.00, 0.31)	0.06
Time, years	16	0.11 (0.00, 0.44)	0.46
Year of publication	18	0.00 (0.00, 0.00)	0.06
**Mild/Moderate Hypoglycaemia**			
Mean age, years	6	0.00 (0.00, 0.00)	0.81
% Male participants	4	0.00 (0.00, 0.01)	0.75
Mean HbA1c, %	6	0.07 (0.00, 0.41)	0.63
Time, years	7	0.00 (0.00, 0.56)	0.28
Year of publication	8	0.00 (0.00, 0.04)	0.28
**Severe Hypoglycaemia**			
Mean age, years	31	0.00 (0.00, 0.00)	0.98
% Male participants	25	0.00 (0.00, 0.00)	0.18
Mean HbA1c, %	18	0.03 (0.00, 0.07)	0.12
Time, years	39	0.00 (0.00, 0.01)	0.41
Year of publication	40	0.00 (0.00, 0.01)	0.41

Abbreviations: CI, Confidence Interval; HbA1c, glycated haemoglobin.

### Incidence of hypoglycaemic episodes per person-year

The pooled incidence of hypoglycaemic episodes per person-year for mild/moderate and severe hypoglycaemia was 19.03 (95% CI 0.00 to 51.08) and 0.80 (95% CI 0.00 to 2.15, Table 2), respectively. Unspecified hypoglycaemia episodes showed a pooled incidence of 27.78 (95% CI 0.00 to 58.20; per person–year). Those on insulin experienced 23.31 (95% CI 0.00 to 58.98) mild/moderate and 1.05 (95% CI 0.00 to 3.69) severe episodes per person-year. Data were not available to calculate any further treatment categories for mild/moderate, but a further incidence of 0.01 (95% CI 0.00 to 0.55) for those on sulphoylureas experiencing severe episodes was estimated.

### Publication bias

There appeared to be publication bias for studies reporting prevalence of severe hypoglycaemia (p = 0.04), with studies reporting lower prevalence appearing to be missing from published literature (funnel plot can be found in S2 Fig). However, when analysis was carried out separating the study results relating to medical assistance only, from any third party assistance, there was no evidence of publication bias (p = 0.41 and; p = 0.36 respectively). No other significant publication bias was shown for prevalence (mild/moderate p = 0.09, unspecified p = 0.37) or for incidence (mild/moderate p = 0.06, severe p = 0.91, unspecified p = 0.06).

## Discussion

This review of 46 studies (n = 532,542) estimates that the prevalence (proportion of people) of hypoglycaemia is 45% for mild/moderate and 6% for severe in population-based studies of type 2 diabetes, and that on average an individual with T2DM experiences 19 mild/moderate episodes and 0.8 severe episodes per year. Hypoglycaemia is particularly prevalent amongst those on insulin (mild/moderate: prevalence = 52%, incidence = 23 events/ year; severe: prevalence = 21%, incidence = 1 event/year, yet still fairly common for treatment regimens that include sulphonylureas (mild/moderate: prevalence = 33%, incidence = 1.92 events/year; severe: prevalence = 5, incidence = 0.01 events/year. Severe hypoglycaemia prevalence was the same 5% for those on treatment regimens that did or did not include sulphonylureas.

### Relationship to other literature

Previous literature focusing on hypoglycaemia in type 2 diabetes in a real-world setting is limited. One report of severe hypoglycaemia prevalence in clinical trials also considered severe hypoglycaemia prevalence in population based studies, but did not examine mild/moderate episodes nor provide pooled estimates [3]. Various systematic reviews have considered hypoglycaemia prevalence within randomised controlled trials involving people with type 2 diabetes [25–28]. These tend to focus on individuals using insulin and their results indicate that severe hypoglycaemia prevalence is below 1%, which is substantially lower than our pooled estimate of 6% from population-based studies. Mild/moderate hypoglycaemia is reported less in clinical trials, though recent meta-analyses of clinical trials showed pooled prevalences of 10% (range 0.7%- 22%) for those on sulphonylureas [34], and 20% to 52% for those on insulin [35–37]. Our estimate for sulphonylureas was higher at 33% (range 30%- 39%), but for insulin was similar, albeit at the higher end of the scale, at 52% (range 20%- 72%). Mild/moderate hypoglycaemia prevalence was fairly consistent across trials, as was the case in the population-based studies in this review. Randomised controlled trials often do not reflect real life situations as treatment regimens are more aggressively titrated than in standard clinical practice, and participants are often highly selected and generally do not include those at higher risk of hypoglycaemia. Therefore, the generalizability of results from trials is limited.

### Strengths and Limitations

To our knowledge, this is the first systematic review and meta-analysis published which focuses on population-based studies of hypoglycaemia in people with type 2 diabetes. The review adhered to the Cochrane recommended standards for systematic reviews [32], involved robust methodology such as duplicate reviewing, and only included population-based studies which reported prevalence and/or incidence rates specifically for a type 2 diabetes population, making the results applicable to the type 2 diabetes general population. The results follow the American Diabetes Association Workgroups recommendations that both the proportion of patients experiencing hypoglycaemia and event rates for each severity are reported[38].

There was high heterogeneity between studies, which was not explained by any of the study level covariates considered. Therefore, the high heterogeneity is likely to be due to the very narrow confidence intervals associated with the proportion estimates, or to other study characteristics which were not measured, reported or extracted. Publication bias appeared to be a possibility for the published studies on severe hypoglycaemia. However, this appeared to be due to the different definitions of severe hypoglycaemia, since no such bias was present when studies that defined severe as requiring medical assistance were separated from those using the standard definition (requiring any third party assistance).

A strict consensus definition was not found across studies (Table 2). Due to the nature of a large proportion of the studies (questionnaire/interview/medical records), a biochemical definition was rarely used. It must be acknowledged that this does create some difficulty when collating and combining results across studies. For some studies this may have caused under or over reporting. Subsequently, the only way to define and analyse hypoglycaemia was by severity and whether or not third party assistance was required during an episode.

The way in which studies categorised different glucose lowering therapies also varied considerably, if indeed they reported hypoglycaemia by treatment categories at all. Therefore, it was only possible to use broader treatment categories in these analyses. It is important to note here also, that the event rate calculated for severe hypoglycaemia in those using sulphonylureas may appear particularly low. This is likely to be due to two of the three studies used to calculate this result only measuring “very severe” hypoglycaemia, those that specifically needed emergency treatment.

Additionally, studies varied considerably in length of time hypoglycaemia prevalence was related to; as a result a specific time period for prevalence could not be established. This was particularly the case for severe hypoglycaemia. However, a meta-regression was carried out for time hypoglycaemia was measured over on prevalence and it was not found to significantly affect the prevalence reported between studies.

Data from some studies could not be grouped by the severity of hypoglycaemia (mild/moderate or severe). These data were therefore labelled as “unspecified” and assumed to include any type of hypoglycaemic episodes. Nocturnal hypoglycaemia was not analysed due to the lack of reporting within studies.

### Implications for practice

This review shows that hypoglycaemia is considerably prevalent amongst people with type 2 diabetes. We estimated that an individual with type 2 diabetes experiences one severe episode of hypoglycaemia per year on average. Severe episodes are a burden on both the individual and healthcare utilisation, due to their cost and the significant dangers that can result from an episode [1, 3]. The estimate of 19 mild/moderate episodes on average per year is also important. This quantity of mild/moderate episodes could substantially impact on work, social life and driving, as well as potentially decreasing general quality of life and increasing risk of severe events if left untreated [39, 40].

Our results highlight an urgent need for raising awareness within everyday clinical practice, particularly as prior evidence has suggested underreporting within this setting [41]. When considering treatment options, hypoglycaemia risk consideration should be incorporated through the individualisation of treatment regimens prescribed [9]. Blood glucose targets should also be individualised and in some cases a higher target may be optimal for the patient [42].

Educational programmes should be focused on successfully increasing knowledge of hypoglycaemia in relation to appropriate self-treatment methods, risk factors and predisposing symptoms [43], as this has previously been shown to be low in the type 2 diabetes population [44, 45].

### Future directions

Research into mild episodes is more challenging than severe episodes in terms of reliability and access to data, with data collection methods limited to constant glucose monitoring, prospective diary recording, and retrospective recall. Constant glucose monitoring is the most reliable, but can be costly and so generally involve small populations and short data collection periods[46, 47]. Retrospective recall is convenient and least costly, though potentially less reliable.

In some studies (6/46, 13%) severity of hypoglycaemia was not specified when data were collected, and hypoglycaemia definitions also varied across studies (Tables 1 and 2). This could have led to errors in reporting, including participants interpreting the meaning of hypoglycaemia differently, with some perhaps only reporting severe episodes. Therefore, it is important that a standardised definition such as the widely used American Diabetes Association workgroup’s[38] is utilised across studies of hypoglycaemia.

More research is needed in relation to potential risks associated with particular glucose lowering therapies. Studies were inconsistent in how they reported treatment regimens, making it difficult to collate data. There is a lack of published population-based literature comparing sulphonylurea and non-sulphonylurea treatment regimens, including the newer glucose lowering therapies.

## Conclusion

To our knowledge, this is the first systematic review and meta-analysis of the prevalence and incidence rates of hypoglycaemic events in population-based studies of people with type 2 diabetes. Hypoglycaemia is particularly prevalent amongst those on insulin, yet still fairly common for treatment regimens that do and do not include sulphonylureas. This highlights the subsequent need for educational interventions and individualisation of therapies to reduce the risk of hypoglycaemia. The current evidence also suggests that hypoglycaemia prevalence within clinical trials is likely to be an underestimate.

## Supporting Information

S1 Fig Search strategy for Ovid MEDLINE. (PDF)

S2 Fig Funnel plot of population based studies reporting severe hypoglycaemia prevalence in type 2 diabetes. (PDF)

S1 Table Quality assessment results table. (PDF)
