# Supplementary material for: Prevalence and Incidence of Hypoglycaemia in 532,542 People with Type 2 Diabetes on Oral Therapies and Insulin: A Systematic Review and Meta-Analysis of Population Based Studies
Source: PLoS One. 2015 Jun 10;10(6):e0126427. doi: 10.1371/journal.pone.0126427 (PMC4465495; doi:10.1371/journal.pone.0126427)
Supplement: S1 Fig — (PDF) [file pone.0126427.s001.pdf]

S1 Fig: Search strategy for Ovid MEDLINE

1. Diabetes Mellitus, Type 2/

2. (type adj "2" adj diabet$).ti,ab.

3. (diabet$ adj type adj (type adj "2" adj diabet$)).ti,ab.

4. (diabet$ adj6 (type adj3 (type adj "2" adj diabet$))).ti,ab.

5. (type adj3 (type adj "2" adj diabet$) adj6 diabetes).ti,ab.

6. 1 or 2 or 3 or 4 or 5

7. Hypoglycemia/

8. Incidence/

9. Prevalence/

10. 8 or 9

11. 7 and 10

12. (hypoglyc$ adj4 prevalence).ti,ab.

13. (hypoglyc$ adj4 incidence).ti,ab.

14. (hypoglyc$ adj4 rate$).ti,ab.

15. (hypoglyc$ adj4 occurrence$).ti,ab.

16. (hypoglyc adj4 frequen$).ti,ab.

17. (hypoglyc$ adj4 event$).ti,ab.

18. (hypoglyc$ adj4 episode$).ti,ab.

19. (hypoglyc$ adj4 risk$).ti,ab.

20. (hypoglyc$ adj4 frequen$).ti,ab.

21. 11 or 12 or 13 or 14 or 16 or 17 or 18 or 19 or 20

22. 21 and 6

23. animal/ not (animal/ and human/)

24. 22 not 23

25. limit 24 to english language

26. "review"/

27. 25 not 2
