# Supplementary material for: Prevalence and Incidence of Hypoglycaemia in 532,542 People with Type 2 Diabetes on Oral Therapies and Insulin: A Systematic Review and Meta-Analysis of Population Based Studies
Source: PLoS One. 2015 Jun 10;10(6):e0126427. doi: 10.1371/journal.pone.0126427 (PMC4465495; doi:10.1371/journal.pone.0126427)
Supplement: S2 Fig — (PDF) [file pone.0126427.s002.pdf]

S2 Fig

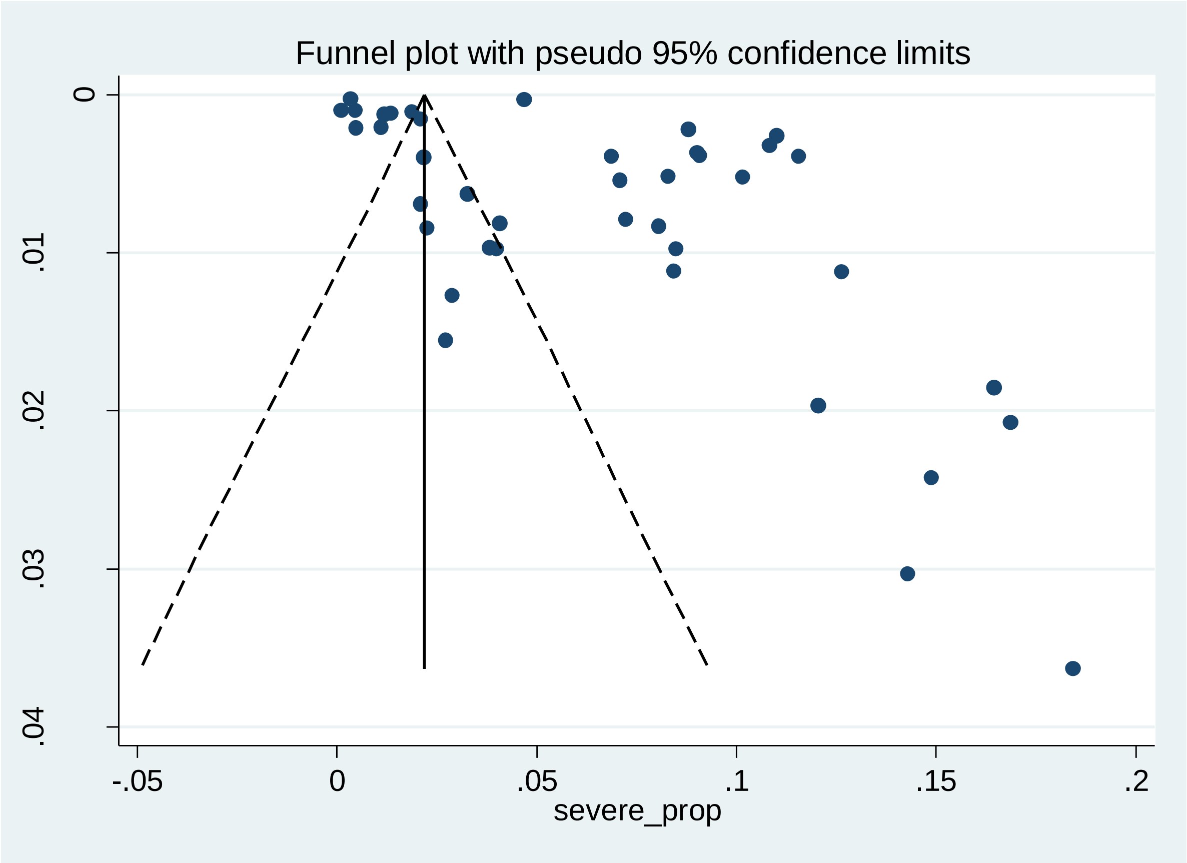

S2 Fig: Funnel plot of population based studies reporting severe hypoglycaemia prevalence in type 2 diabete
