# Supplementary material for: Prevalence and Incidence of Hypoglycaemia in 532,542 People with Type 2 Diabetes on Oral Therapies and Insulin: A Systematic Review and Meta-Analysis of Population Based Studies
Source: PLoS One. 2015 Jun 10;10(6):e0126427. doi: 10.1371/journal.pone.0126427 (PMC4465495; doi:10.1371/journal.pone.0126427)
Supplement: S1 Table — (PDF) [file pone.0126427.s003.pdf]

S3 Table: Quality assessment results table

	Sample Bias					Data Collection			Confounding factors
**Author, Year**	**Sample source representative and described well**	**Sampling method**	**Eligibility criteria Applied and described**	**Sufficient sample response**	**Overall for Sample Bias**	**Data collection tool well described**	**Measurement reliability**	**Overall for Data Collection**	**Consideration of confounding/explanatory factors**
Akram, 2006	+	-	++	+	+	++	+	++	++
Allen, 2004	+	-	++	-	-	++	++	++	++
Andel, 2008	++	-	++	-	-	++	+	++	-
Aung, 2011	++	+	++	-	+	++	+	++	++
Bourdelmarch-asson, 2007	++	+	++	-	+	++	+	++	++
Chan, 2010	++	-	++	-	-	++	+	++	++
Davis, 2005	++	++	++	-	+	++	+	++	++

Davis, 2010	++	-	++	+	+	++	++	++	++
Donnelly, 2004	++	+	++	-	+	++	++	++	++
Green, 2012	++	++	++	+	++	++	+	++	++
Gurlek, 1999	+	-	++	-	-	+	+	+	++
Henderson, 2003	+	+	++	-	+	++	+	++	++
Holstein, 2003	++	++	++	++	++	++	++	++	++
Honkasalo, 2011	++	++	++	+	++	++	+	++	++
Jaap, 1998	++	-	++	-	-	++	+	++	++
Johnston, 2012	++	++	++	++	++	++	++	+	++
Katon, 2013	++	++	++	+	++	++	++	++	++
Krnacova, 2012	++	++	+	++	++	++	++	++	++
Lecomte, 2008	++	+	++	-	+	++	+	++	++
Leese, 2008	++	++	++	++	++	++	++	++	++
Leiter, 2005	++	-	++	++	+	++	+	++	++
Lin, 2012	++	+	++	++	++	++	++	++	++
Lipska, 2013	++	++	++	+	++	++	+	++	++
Lundkvist, 2005	++	-	++	-	-	++	+	++	++

Maggi, 2013	++	-	++	-	-	+	+	+	++
McCoy, 2012	+	-	++	-	-	++	+	++	++
McCoy, 2013	++	++	++	-	+	++	+	++	++
Miller, 2001	+	++	++	++	++	++	+	++	++
Murata, 2004	++	+	++	-	+	++	++	++	++
Neil, 2007	++	++	++	-	++	++	+	++	++
Ooi, 2011	++	-	++	-	-	+	+	+	-
Parsaik, 2013	++	++	++	++	++	++	++	++	++
Pettersson, 2010	++	-	++	-	-	++	+	++	++
Rombopoulos, 2013	++	+	+	-	+	++	+	++	++
Samann, 2012	++	++	++	-	+	++	++	++	++
Sarkar, 2010	++	++	++	+	++	++	+	++	++
Schopman, 2009	+	+	++	-	+	++	+	++	++
Skinner, 2013	+	++	++	-	+	++	+	++	++
Stahl, 1999	++	++	++	++	++	++	++	++	++
Stargardt, 2009	++	-	++	-	-	++	+	++	-

Vexiau, 2008	++	-	++	-	-	++	+	++	++
Whitmer, 2009	++	++	++	++	++	++	++	++	++
Williams, 2012	++	+	++	-	+	+	+	+	++
Yun, 2013	+	-	++	+	+	++	+	++	++
The hypoglycaemia study group, 2007	++	++	++	++	++	++	++	++	++
Zhang, 2013	+	-	++	-	-	+	+	+	++

*Criteria were marked on a scale of "++", "+" or "-"
